# Toxicity of glyphosate accelerates neurodegeneration in *Caenorhabditis elegans* model of Alzheimer’s disease

**DOI:** 10.3389/ftox.2025.1578230

**Published:** 2025-07-01

**Authors:** Nisha Rani, Mohammad Mumtaz Alam, Suhel Parvez

**Affiliations:** ^1^ Department of Toxicology, School of Chemical & Life Sciences, Jamia Hamdard, New Delhi, India; ^2^ Department of Pharmaceutical Chemistry, School of Pharmaceutical Education & Research, Jamia Hamdard, New Delhi, India

**Keywords:** *Caenorhabditis elegans*, Alzheimer’s disease, glyphosate, behavioural toxicity, amyloid beta, oxidative stress

## Abstract

**Introduction:**

Pesticide-related environmental contamination poses a growing global concern, threatening human health, wildlife, and ecosystems. Glyphosate (N-phosphonomethyl-glycine, GLY), a widely used organophosphorus herbicide, has been associated with neurotoxic effects. This study investigates the potential neurodegenerative impact of glyphosate using a Caenorhabditis elegans Alzheimer’s disease (AD) model.

**Methods:**

Transgenic *C. elegans* strain CL4176, which expresses human amyloid-beta (Aβ1–42) upon temperature induction, was exposed to various concentrations of glyphosate (12, 15, 18.5, 20, and 25 mg/L) for 24 hours. Behavioral assays (body bends, head thrashes, body length, and pharyngeal pumping), oxidative stress markers (catalase activity), and Aβ protein expression were evaluated.

**Results:**

Glyphosate exposure induced a concentration-dependent decline in locomotor and feeding behaviors. Catalase activity was significantly reduced, indicating elevated oxidative stress. Additionally, a marked increase in Aβ1‐42 protein expression was observed in glyphosate-treated CL4176 worms.

**Discussion:**

These findings suggest that glyphosate exacerbates Aβ toxicity and induces AD-like phenotypes in the *C. elegans* model through behavioral impairment, oxidative stress, and increased Aβ accumulation. Glyphosate’s potential contribution to neurodegenerative processes warrants further investigation.

## 1 Introduction

Neurodegenerative diseases are becoming increasingly common, yet their causes remain largely unclear, and current treatments are mostly not effective (Chen et al., 2015). Alzheimer’s disease (AD), a chronic neurodegenerative condition, poses a significant threat to individual health. The pathogenic mechanisms of Alzheimer’s disease (AD) have been extensively studied over the years. Several hypotheses have been put up, including oxidative stress, amyloid toxicity, hyperphosphorylated tau protein, and cholinergic effects. Among these, the amyloid toxicity hypothesis is the most widely accepted. Amyloid-beta (Aβ) peptides, specifically Ab1-40 and Aβ1-42, are formed when the amyloid precursor protein (APP) is cleaved abnormally (Yu et al., 2024). These peptides then build up in neurons. Aβ1-42, in particular, has been recognized as a major mediator of oxidative stress, playing a central role in AD pathogenesis by decreasing levels of superoxide dismutase (SOD) and raising levels of malondialdehyde (MDA) and reactive oxygen species (ROS) (Butterfield and Boyd-Kimball, 2004; Wang et al., 2021). Due to its central role in the disease, amyloid is frequently targeted in drug screening and clinical diagnostics.Pesticides encompass a wide range of chemicals intentionally released into the environment to control undesirable insects, plants, fungi, and other organisms (Hernández et al., 2013). Pesticides are classified into insecticides, herbicides, fungicides.Among these, herbicides containing glyphosate as the active ingredient are the most extensively used worldwide (Garud et al., 2024). Glyphosate (N-phosphonomethyl-glycine) (GLY), known for its high solubility in water, is an organophosphorus compound. Studies have shown that organophosphorus compounds, including GLY, can induce neurotoxic effects in rats, fish, etc., models (Burns et al., 2013; Ait-Bali et al., 2020). Accidental intoxication with GLY has been allied with neural symptoms such as ataxia, tingling, tremors, loss of coordination,and neuropathy (Pereira et al., 2018). Using both *in vivo* and *in vitro* tests on various organisms, such as *C. elegans*, rats, and fish, the toxicological profile of GLY has been investigated (Costas-Ferreira et al., 2022). These studies have revealed significant impairmentsin exposed adults and/or offspring, particularly cytotoxicity in glutamatergic, cholinergic, GABAergic, and dopaminergic neurons (Pereira et al., 2018; Jacques et al., 2019). In the past few years, *Caenorhabditis elegans* has become a vital animal model for toxicological and environmental studies, both in entire animals and individual cells. This organism offers several advantages, such as a transparent body, short life cycle, easy handling, and well-established genetic and molecular backgrounds (Leung et al., 2008; Xu et al., 2017). Additionally, *C. elegans* is highly sensitive to environmental toxicants, making it particularly useful for toxicology research (Liu et al., 2022). With its 302 neurons and fully mapped neuronal lineage, *C. elegans* is an excellent model for neurotoxicology studies (Wu et al., 2014; Xu et al., 2017). The lack of a functioning blood-brain barrier allows chemical molecules to rapidly diffuse into thenervous system upon uptake (Nava-Ruiz et al., 2012). As a result, *C. elegans* has been recognized as a significant alternative model for toxicity evaluation and the study of underlying mechanisms (Xu et al., 2017).In the present study,we aimed to explore the effects of Glyphosate on mortality, growth, biophysiological and behavioural activities in the transgenic *C. elegans* model.

## 2 Material and methods

### 2.1 Chemicals and reagents used

NaCl, dextrose, KH_2_PO_4_, cholesterol, KOH, NH_4_Cl were procured from SRL chemicals (Mumbai, India). Bacto-agar was purchased from Becton, Dickinson, and Company (France). CaCl_2_, Na_2_HPO_4_, and MgSO_4_ were purchased from Merck (Mumbai, India). Peptone was produced by HiMedia Laboratories Pvt. Ltd. (Mumbai, India). Uracil was procured from Sigma-Aldrich, China. Sigma-Aldrich, United States provided glyphosate. reagents and chemicals usedwere highly pure (99.9%).

### 2.2 *Caenorhabditis elegans* strains and maintenance

The transgenic *C. elegans* CL4176 strain [smg-1 (myo-3/Aβ-1-42 long 3′-untranslated region (UTR)] and the wild type *C. elegans* N2 (Bristol) strain were obtained from Central Drug Research Institute, Lucknow. The wild-type N2 strain was maintained at 20°C, whereas the CL4176 strain was maintained at 16°C. The CL4176 transgenic strain expresses a body wall muscle specific Aβ depending on raising the ambient temperature from 16°C to 23°C to induce Aβ expression until adulthood) (Zamberlan et al., 2014). According to a standard protocol, all worms were grown on nematode growth medium (NGM) plates containing NaCl, Peptone, Bacto-agar, Cholesterol, MgSO_4_, CaCl_2_, and KH_2_PO_4_ seeded with 300 µL lawn of *Escherichia coli* (OP50) strain as a food source. To prepare age-synchronized worms, nematodes were transferred to new NGM plates, and the eggs were obtained by isolating embryos from gravid hermaphrodites and treating the worms with 5% bleach solution and NaOH solution (Zamberlan et al., 2014). The synchronized L4-larval stage was isolated and cultured on fresh NGM plates in either 20°C or 16°C (for N2 and CL4176 strains).

### 2.3 Lethal toxicity test

To assess the toxicodynamic effect of glyphosate in *C. elegans* strains, a synchronized L4 stage of wild type N2 and CL4176 transgenic strain were transferred to NGM plates containing various concentrationsof glyphosate in 12-well plates for 24 h at 20°C and 16°C. Each well contained about 30 worms with food. Five concentrations of GLY were tested in three replicates. The tested concentrations were between 12 mg/L and 25 mg/L compared with *C. elegans* wild type N2 control and *C. elegans* AD strains. A platinum wire was used to probe the worms over the NGM plate, and then the number of living worms was observed using an LMI stereo zoom microscope (Wang et al., 2017).

### 2.4 *Caenorhabditis elegans* behavioral toxicity analysis

Synchronized L4 population of wild-type N2 and transgenic worms (CL4176) were exposed to a sublethal dose of glyphosateand incubated for 24 h at 20°C and 16°C. After the worms were collected and washed three times in M9 buffer, the body bend rate and head thrashing rate were assessed using the 1/2nd and 1/5^th^concentrations of LC_50_ of glyphosate in both the strains (Mishra and Agarwal, 2020).

#### 2.4.1 Head thrashing behavior

In order to examine the head thrashing rate, worms were isolated from NGM plates with sublethal concentrations of GLY and washed the wormswith M9 buffer solution. After treatment, washed worms were transferred into new NGM plates without food. After 2 min of resting period, thrashes produced by worms were counted for 1 min. In each group, 8-10 worms were examined in three independent experiments. Head thrash is defined as the change in the mid-body’s bending direction.

#### 2.4.2 Body bends behavior

To evaluate the body bend behavior, worms were isolated from NGM plates having sublethal concentrations of GLY and transferred into new NGM plates without food. They were recorded for the Body bends produced in a 20s time interval following a 2 min adaptation period. In each group, 8-10 worms were examined in triplet form of experiment. A body bend was defined as a change in the direction of propagation of the part of the worm corresponding to the posterior bulb of the pharynx along the *y*-axis, assuming the worm was traveling along the *x*-axis. The dysfunction and eventually complete loss of motor neuron activity due to exposure to toxicants, such as glyphosate and other pesticides, is the most plausible explanation for the body bend behavior.

### 2.5 Pharyngeal pumping behavior

To determine the pharyngeal pumping behaviour, worms were isolated from NGM plates with sublethal glyphosate concentrations. They with sublethal concentrations of glyphosate, and the worms were washed with M9 buffer solution. After the toxicant exposure, worms were transferred into new NGM plates with food. After 2 min of the resting period, the number of pumps was counted as 30s. The experiment was repeated thrice.

### 2.6 Body length assay

For body length assay, a series of sublethal doses of glyphosate (12, 15, 18.5, 20, and 25 mg/L) were exposed to worms (L4 stage) of both strains for 24 h in 12-well plates at 22°C and 16°C respectively. Worms were placed onto new NGM plates after being treated, washed in M9 buffer, and transferred onto new NGM Petri plates. Using 0.1% sodium azide, the worms were paralyzed and mounted on a glass plate. The body length of worms was measured by stereo zoom microscope and analyzed by ImageJ software. In each group, 8-10 worms were measured in three independent experiments.

### 2.7 Lipid peroxidation assay

The term ”Lipid peroxidation” describes the oxidative breakdown of lipids and is a sign of oxidative stress in tissues and cells. Age-synchronized L4 worms of wild-type N2 and CL4176 AD strains were exposed to sublethal doses of glyphosate in 12-well plates for 24 h at 20°C and at 16°C. Approximately 4,000–5,000 worms were isolated and washed three times with M9 buffer. The worms were homogenized individually in chilled phosphate buffer (0.1 M, pH-7.4), 0.250 mL of homogenate was mixed with 0.025 mL of 10 mM BHT (butyl-hydroxytoulene) and 1 mL of 0.67% TBA (thiobarbituric acid). After vertexing, 3 mL of 1% OPA (o-phosphoric acid) was added. The reaction mixture was incubated at 90°C in a boiling water bath for 45 min. After removal, the mixture was allowed to cool at ambient temperature. The intensity of pink chromogen that was produced was measured at 535 nm. The lipid peroxidation rate during the reaction was calculated as nanomoles of thiobarbituric acid reactive substance (TBARS) formedusing a molar extinction coefficient of 1.56 × 10^5^ M^-1^cm^-1^.

### 2.8 Catalase activity assay

Synchronized L4 larval worms of wild-type N2 and CL4176 AD strains were exposedtosublethal glyphosate concentrations in 12-well plates for 24 h at 20°C and16°C. About 4,000 worms were isolated and washed three times with M9 buffer. 10% homogenate of worms was prepared in chilled phosphate buffer (0.1M, pH-7.4), then centrifuged at 10,000 rpm for 12 min at 4°C. The supernatant was collected in another tube and used to determine the catalase activity. Using a spectrophotometer, the rate of evaporation of H_2_O_2_ was measured at 240 nm for 3 min at intervals of 15 s to determine the catalase activity. The enzyme activity was calculated using the molar extinction coefficient of 43.6 M^-1^cm^-1^.

### 2.9 Western blotting

Synchronized L4 larval stage *C. elegans* (n = 5,000/group) were incubated in M9 buffer solution in a 24-well plate, with triplicate sets for the control and three sublethal concentrations of glyphosate. After24 hours after treatment, the worms were collected and washed 3–24 h after treatment, and the worms were collected and washed 3 times in M9 buffer. Whole worm bodies were homogenized in lysis buffer containing a protease inhibitor cocktail, followed by 2 min of sonication on ice to prevent protein degradation. The lysate was centrifuged at 15,000 g for 12 min at 4°C (Protein concentration was calculated by Bradford assay, and an equal amount of protein was loaded for sodium dodecyl sulfate-polyacrylamide gel electrophoresis (SDS-PAGE) analysis. Protein (15–20 µg) was loaded onto 15% SDS-PAGE gels and transferred to a 0.45 μm PVDF (Polyvinylidene fluoride) membrane. Immunoblotting was performed using primary antibodies (Cleaved Caspase-3, beta-Amyloid from Cell Signaling, and β-Actin (reference protein) from Abbkine, 1:1000), followed by secondary antibodies (goat anti-mouse IgG from Abbkine, 1:10,000). ECL chemiluminescence substrate (BioRad) was used for visualization, and densitometric analysis was performed using ImageJ software (Ali et al., 2022; Rehman et al., 2025).

### 2.10 Statistical analysis

All the data are represented as means ± standard deviation. Using probit analysis, the median lethal concentration (LC_50_) was determined. GraphPad Prism version six from GraphPad Software, United States, was used for all the statistical analysis. Statistical significance between control N2, control AD, and treatment groups in thrashing frequency, body bend movement, body length, pharyngeal pumping,and protein expression was analyzedby one-way ANOVA analysis with the Tukey’s Test.

## 3 Results

### 3.1 To evaluate the toxicodynamic effects of glyphosate

**FIGURE 1:**
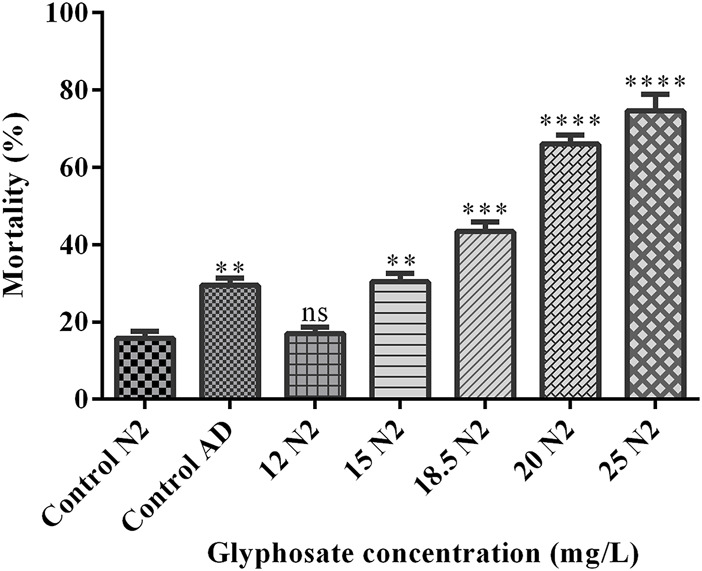
Experimental design of glyphosate toxicity at different doses in *Caenorhabditis elegans* strains.

**FIGURE 2:**
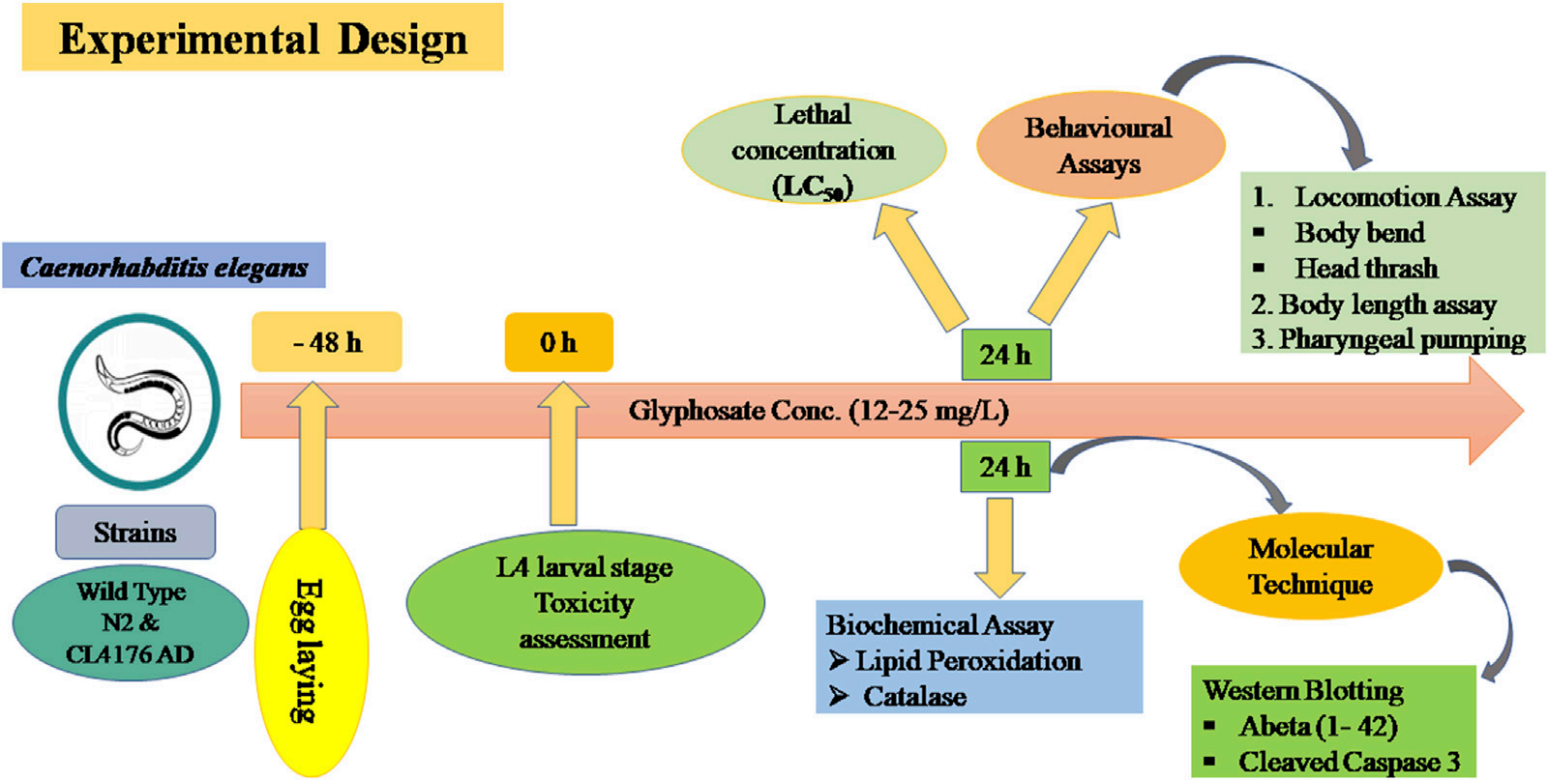
Representative graph shows the effects of glyphosate (GLY) on the *Caenorhabditis elegans* through Lethal concentration. It shows the mortality rate of *Caenorhabditis elegans* exposed to 12, 15, 18.5, 20 and 25 mg/L of glyphosate for 24 h in 24 well plates (n = 3). There was a significant difference in the mortality rate of *Caenorhabditis elegans* at different concentrations of glyphosate compared to Control N2. n = 3 **P < 0.01,***P < 0.001 and ****P < 0.0001. This graph shows the same toxicity rate in the Control AD group and the 15 mg/L group of Control N2. (**P < 0.01).

To investigate the toxicity of glyphosate, we performed a lethal concentration assay to monitor the mortality rate of wild type N2 strain and CL4176 AD strain of *C. elegans*. After treatmentof glyphosate with different concentrations (12–25 mg/L), the mortality percentage of *C. elegans* was raised-dose-dependent. A stereo-zoom dissecting microscope was used to count the live worms of both strains (N2 and CL4176 AD). As shown in Figure 1, LC_50_ (half lethal concentration) was obtained at the concentration of 18.5 mg/L glyphosate. We found that the survival rate of the control AD strain was similar to that of the wild-type N2 strain of *C. elegans* at the concentration of 15 mg/L glyphosate. We used sub-lethal glyphosate concentrations (1/2nd and 1/5th of LC_50_) for further studies and examined different behavioural and molecular toxicity as shown in Figure 2.

### 3.2 Effects of glyphosate on locomotorybehaviors

**FIGURE 3:**
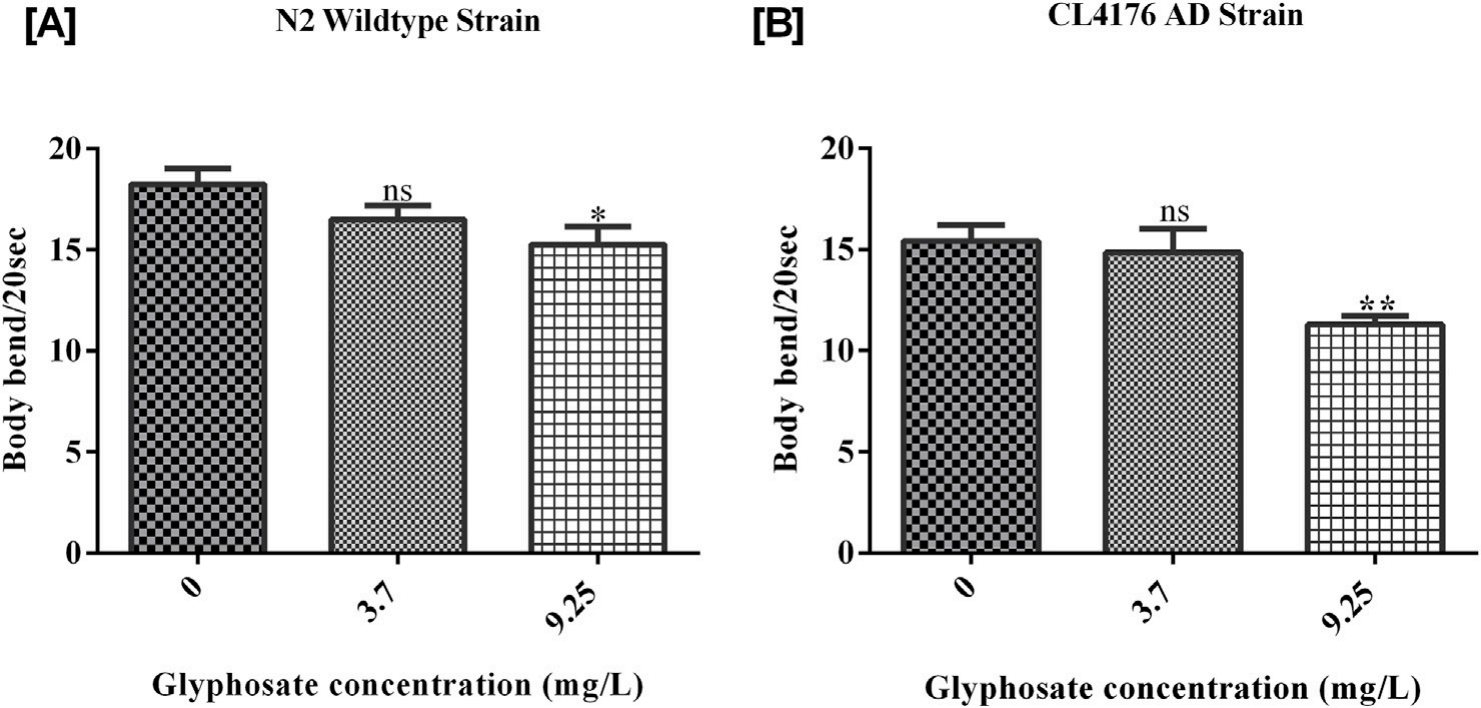
Representative graphs show the effect of glyphosate on the behavior of *Caenorhabditis elegans*. Synchronized N2 wild-type and CL4176 AD worms were treated with different concentrations of glyphosate (3.7and 9.25 mg/L), and the body bend per 20 s was evaluated after 24 h of exposure. **(A)** There was a significant difference between Control N2 and high dose of glyphosate (n = 3) *P < 0.05. **(B)** There was a significant difference between Control AD and high dose of glyphosate (n = 3) **P < 0.01.

**FIGURE 4:**
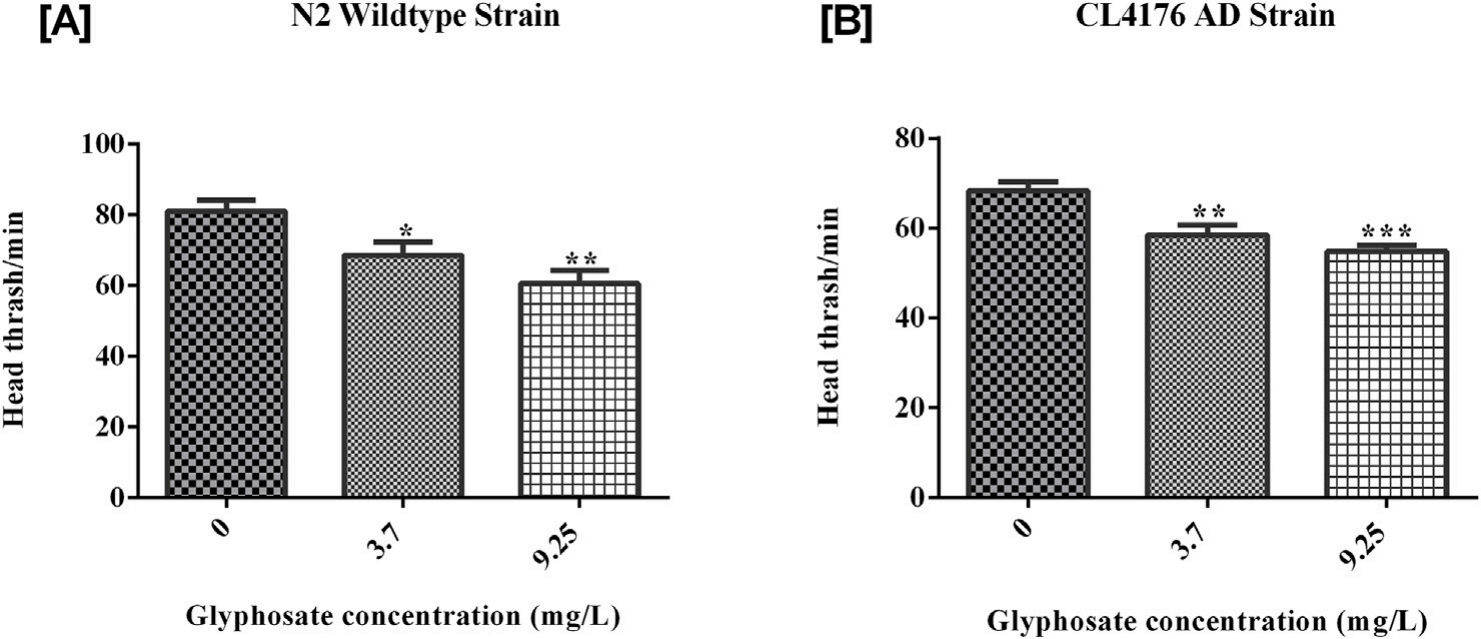
Representative graphs show the effect of glyphosate on the behavior of *Caenorhabditis elegans*. Synchronized N2 wild-type and CL4176 AD worms were treated with different concentrations of glyphosate (3.7and 9.25 mg/L), and the head thrash per 60 secondswasevaluated after 24 h of exposure. **(A)** There was a significant difference in Control N2 with low and high dose of glyphosate (n = 3) *P < 0.05, **P < 0.01. **(B)** There was a significant difference in Control AD with low and high doses of glyphosate (n = 3) **P < 0.01, ***P < 0.001.

To evaluate the toxicity of glyphosate on locomotory behaviors like head thrash and body bend assay, worms of both strains (wild type N2 and CL4176 AD) were treated with sublethal doses of glyphosate for 24 h at 20°C and at 16°C. As shown in Figure 3A, the head thrash of N2 worms was significantly reduced in all sub-lethal concentrations as compared with control N2, and in Figure 3B, the head thrash of CL4176 AD worms was also reduced in all sub-lethal concentrations as compared with control AD. The body bend frequency of worms (wild type N2 and CL4176 AD) asas decreased with increasing the concentrations of glyphosate inadependent manner. As shown in Figure 4A, a significant difference between Control N2 and a high dose of glyphosate was observed. In Figure 4B, a significant difference between Control AD and high doses of glyphosate was found.

### 3.3 Effects of glyphosate on pharyngeal pumping behaviour

**FIGURE 5:**
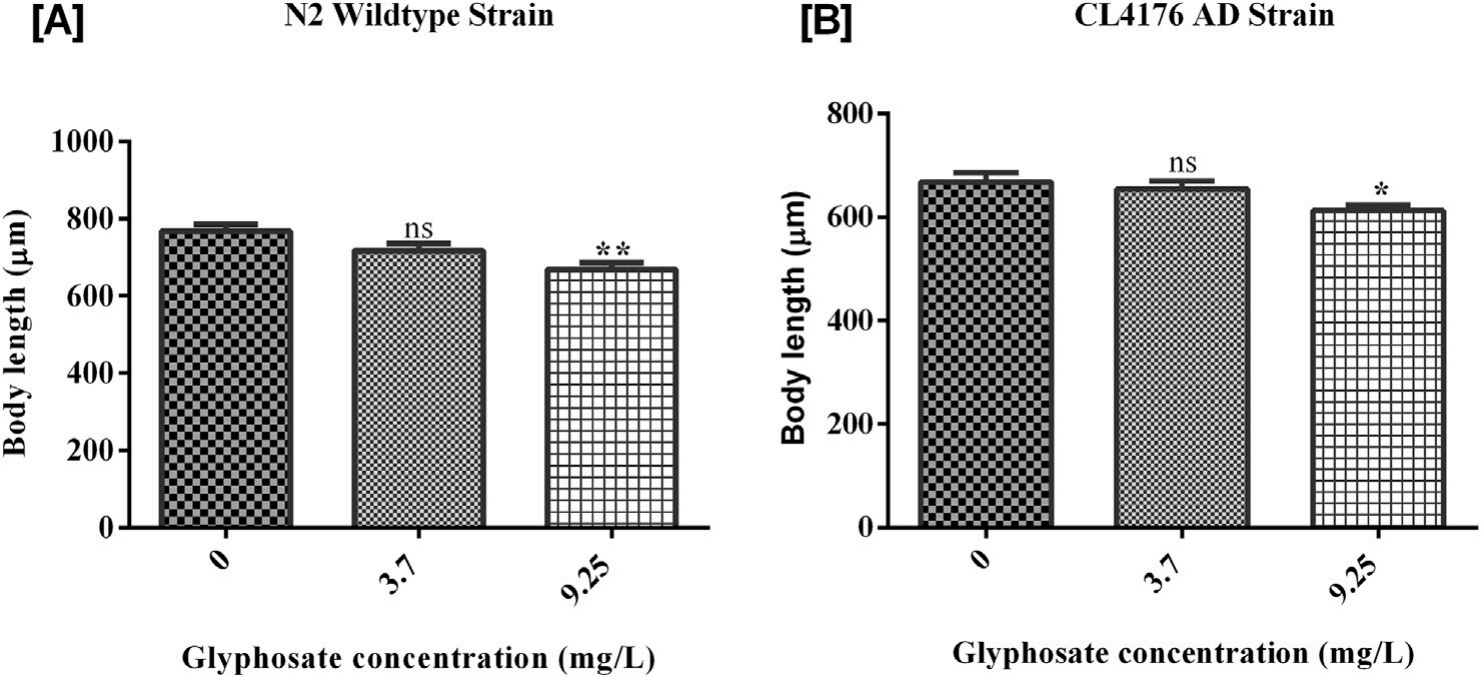
Representative graphs show the effect of glyphosate on the length of *Caenorhabditis elegans*. Synchronized N2 wild-type and CL4176 AD *Caenorhabditis elegans* were treated with different concentrations of glyphosate (3.7and 9.25 mg/L), and the body length wasevaluated after 24 h of exposure. **(A)** There was a significant difference between Control N2 and high dose of glyphosate (n = 3) **P < 0.01. **(B)** There was a significant difference between Control AD and a high dose of glyphosate (n = 3) *P < 0.05.

The pharyngeal pumping of worms treated with the sublethal concentrations of glyphosate was significantly reduced compared to control N2 and control AD worms. Synchronized N2 wild-type and CL4176 AD *C. elegans* were treated with different concentrations of glyphosate, and the pharyngeal pumping was calculated after 24 h of exposure. Results showed in Figure 5A, there was a significant difference between Control N2 and a high dose of glyphosate. In Figure 5B, a significant difference in Control AD was observed with low and high doses of glyphosate.

### 3.4 Effects of glyphosate on the growth of *Caenorhabditis elegans*

**FIGURE 6:**
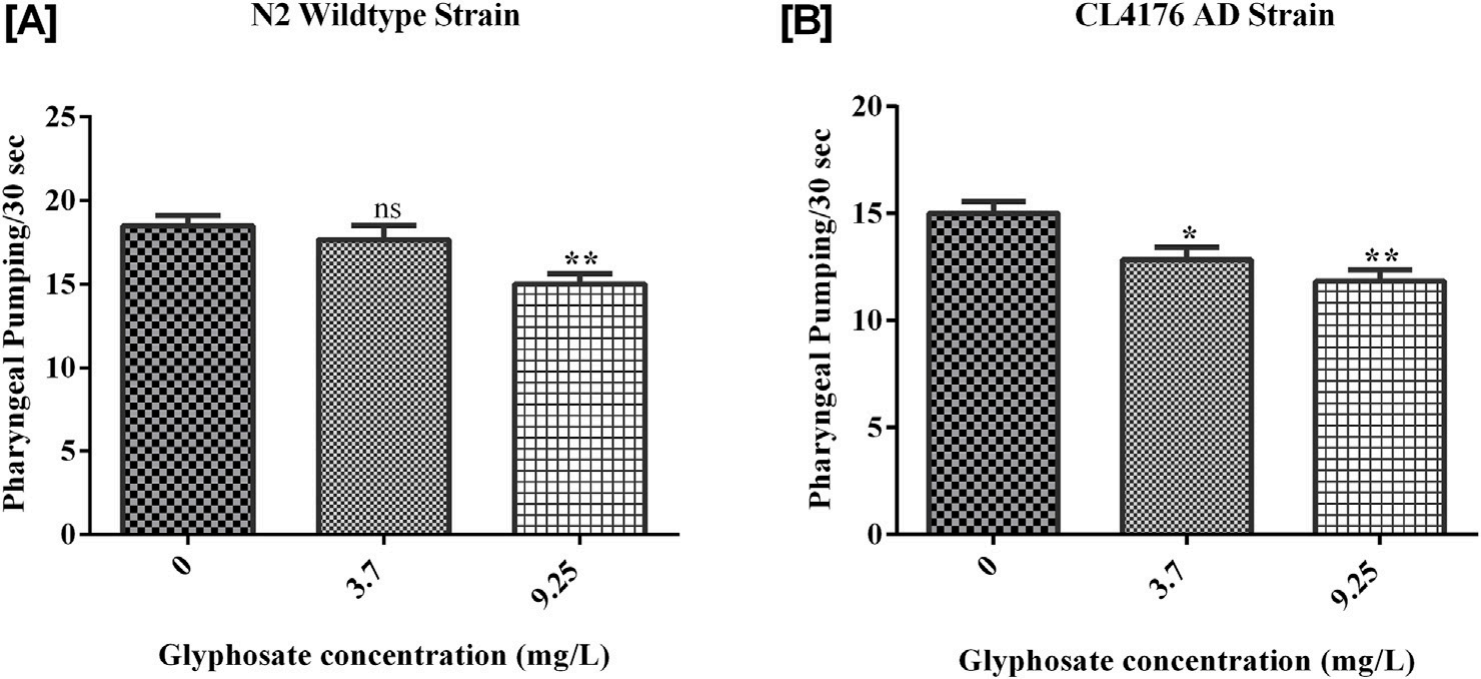
Representative graph shows the effect of glyphosate on pharyngeal pumping of *Caenorhabditis elegans*. Synchronized N2 wild-type and CL4176 AD *Caenorhabditis elegans* were treated with different concentrations of glyphosate (3.7 mg/L, 9.25 mg/L), and the pharyngeal pumping was calculated after 24 h of exposure. **(A)** There was a significant difference between Control N2 and high dose of glyphosate (n = 3) **P < 0.01. **(B)** There was a significant difference in Control AD with low and high doses of glyphosate (n = 3) *P < 0.05, **P < 0.01.

The growth of worms was measured and analyzed after the exposure of glyphosate for 24 h. As shown in Figure 6A, there was a significant difference between Control N2 and a high dose of glyphosate. The worms in the wild-type N2 group that received a high dose of glyphosate showed a significant reduction in body length compared to the untreated control group. [B] There was a significant difference between Control AD and high doses of glyphosate in the AD group. In the Alzheimer’s model strain (AD), the worms exposed to the high dose of glyphosate also had significantly reduced body length compared to their control AD group. There was no significant difference between the control N2 group and the low dose (1/5th of LC_50_) of glyphosate in wild type N2 group. The body length of nematodes was significantly decreased in a high sublethal dose of glyphosate of wild type N2 group and also decreased in all groups (control, low dose, and high dose) of AD strain.

### 3.5 Glyphosate increased oxidative stress in *Caenorhabditis elegans*

**FIGURE 7:**
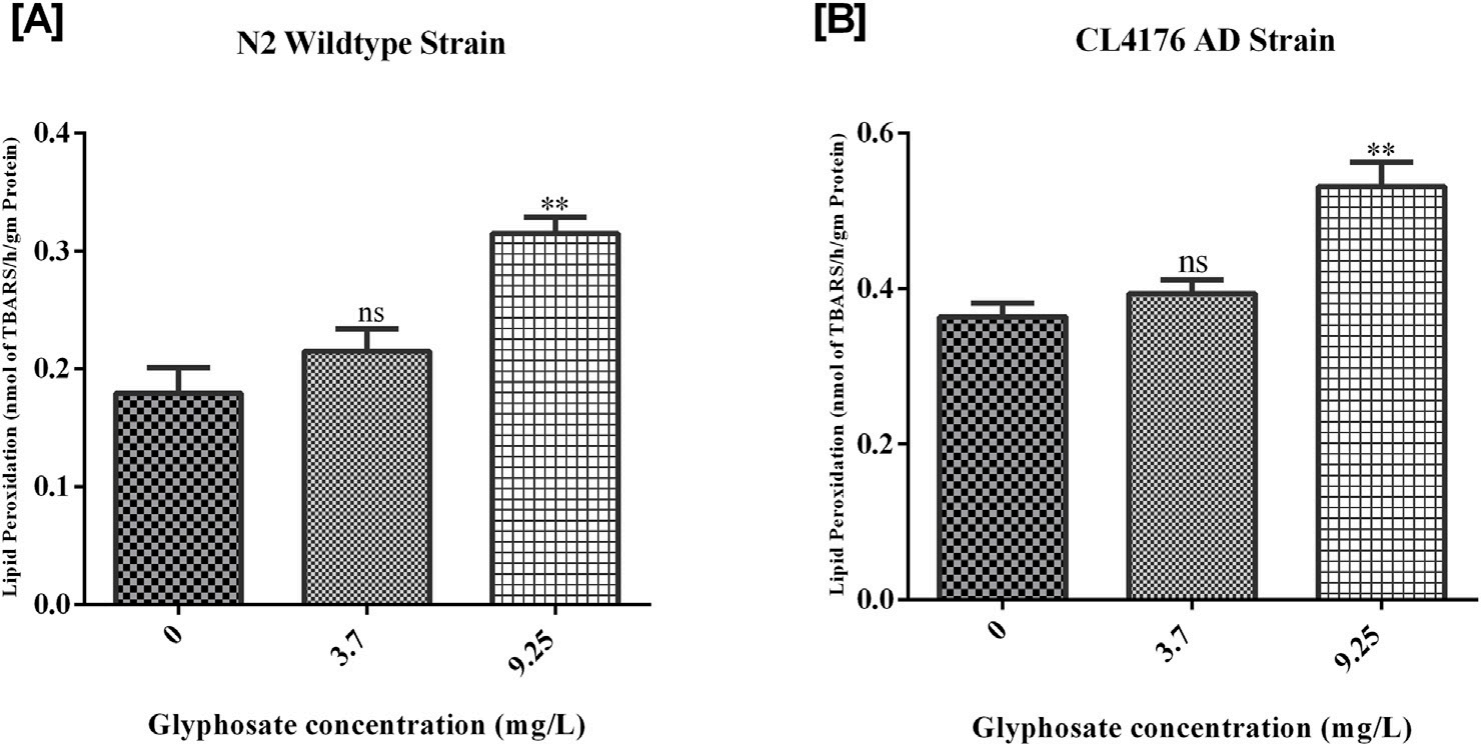
Representative graphs show the effect of glyphosate treatment on oxidative stress in *Caenorhabditis elegans*. Synchronized N2 wild-type and CL4176 AD *Caenorhabditis elegans* were treated with different concentrations of glyphosate (3.7and 9.25 mg/L). **(A)** Lipid peroxidation increases in high doses of glyphosate as compared to Control N2 (n = 3) **P < 0.01. **(B)** Lipid peroxidation increases in high doses of glyphosate as compared to the Control AD group (n = 3) **P < 0.01.

Lipid peroxidation (LPO) was measured as an indicator of oxidative stress. This study found that glyphosate increased TBARS levels in all the treated groups. This rise in TBARS, a breakdown product of membrane lipids, suggests the generation of oxidative stress. As shown in Figure 7A, there was a significant rise in TBARS levels in high doses of glyphosate as compared to Control N2. [B] There was a significant increase in TBARS levelin high doses of glyphosate as compared to Control AD.

### 3.6 Glyphosate decreased catalase activity in *Caenorhabditis elegans*

**FIGURE 8:**
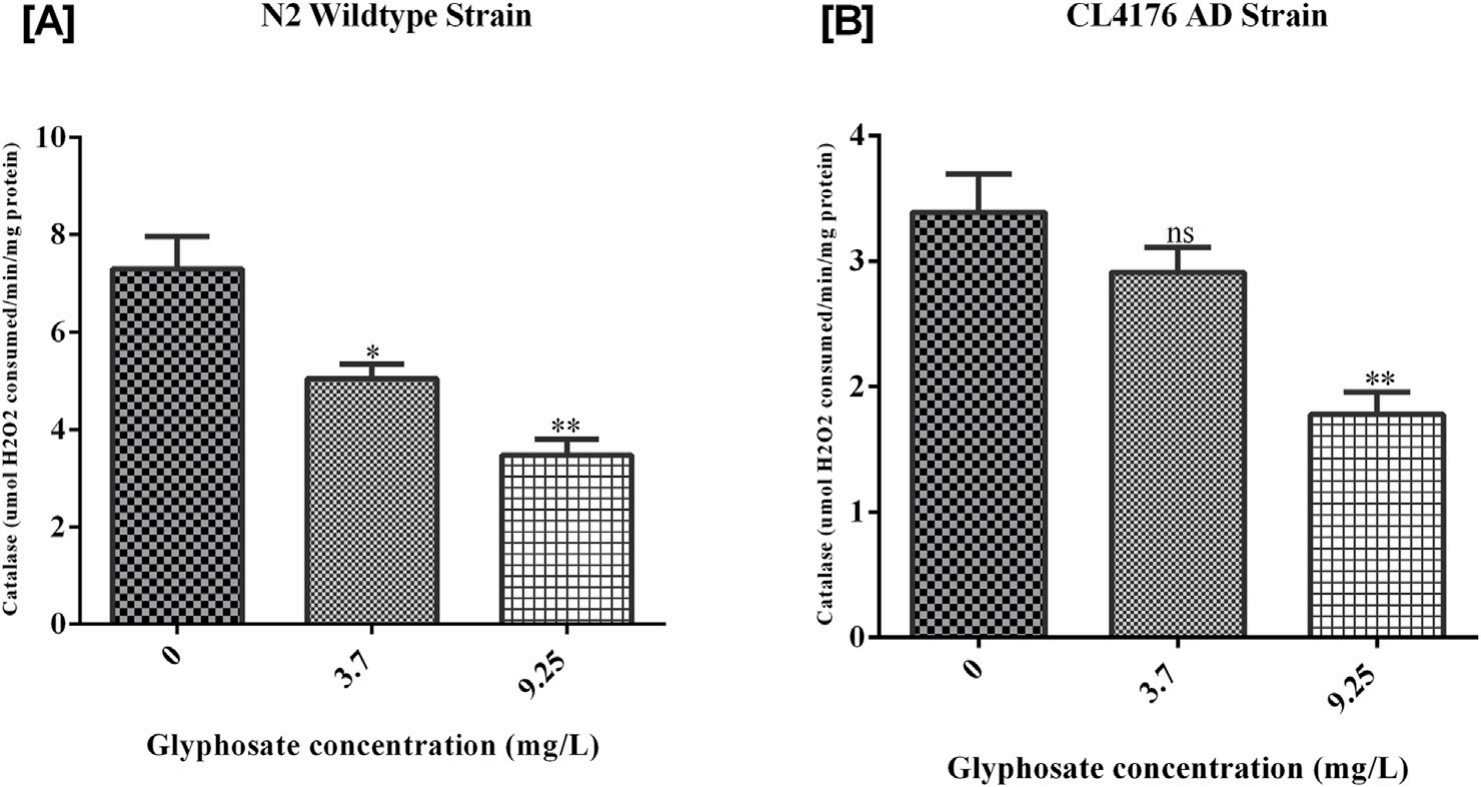
Representative graph shows the effect of glyphosate on catalase (antioxidant) enzyme in *Caenorhabditis elegans.* Synchronized N2 wild-type and CL4176 AD *Caenorhabditis elegans* were treated with different concentrations of glyphosate (3.7and 9.25 mg/L). **(A)** The catalase activity was highest in the Control N2 group. There was a significant reduction in both the treated groups (n = 3) *P < 0.05, **P < 0.01. **(B)** There was a significant reduction of catalase enzyme in high doses of glyphosate as compared to the Control AD group (n = 3) **P < 0.01.

Catalase isan important antioxidant enzyme in *C. elegans*. The catalase activity was decreased with an increase in the glyphosate concentrations. As shown in Figure 8A, the catalase activity was highest in the Control N2 group. There was a significant reduction in both the treated groups. [B] There was a significant reduction of catalase enzyme in high doses of glyphosate compared to Control AD.

### 3.7 Effects of glyphosate on protein markers in *Caenorhabditis elegans*

**FIGURE 9:**
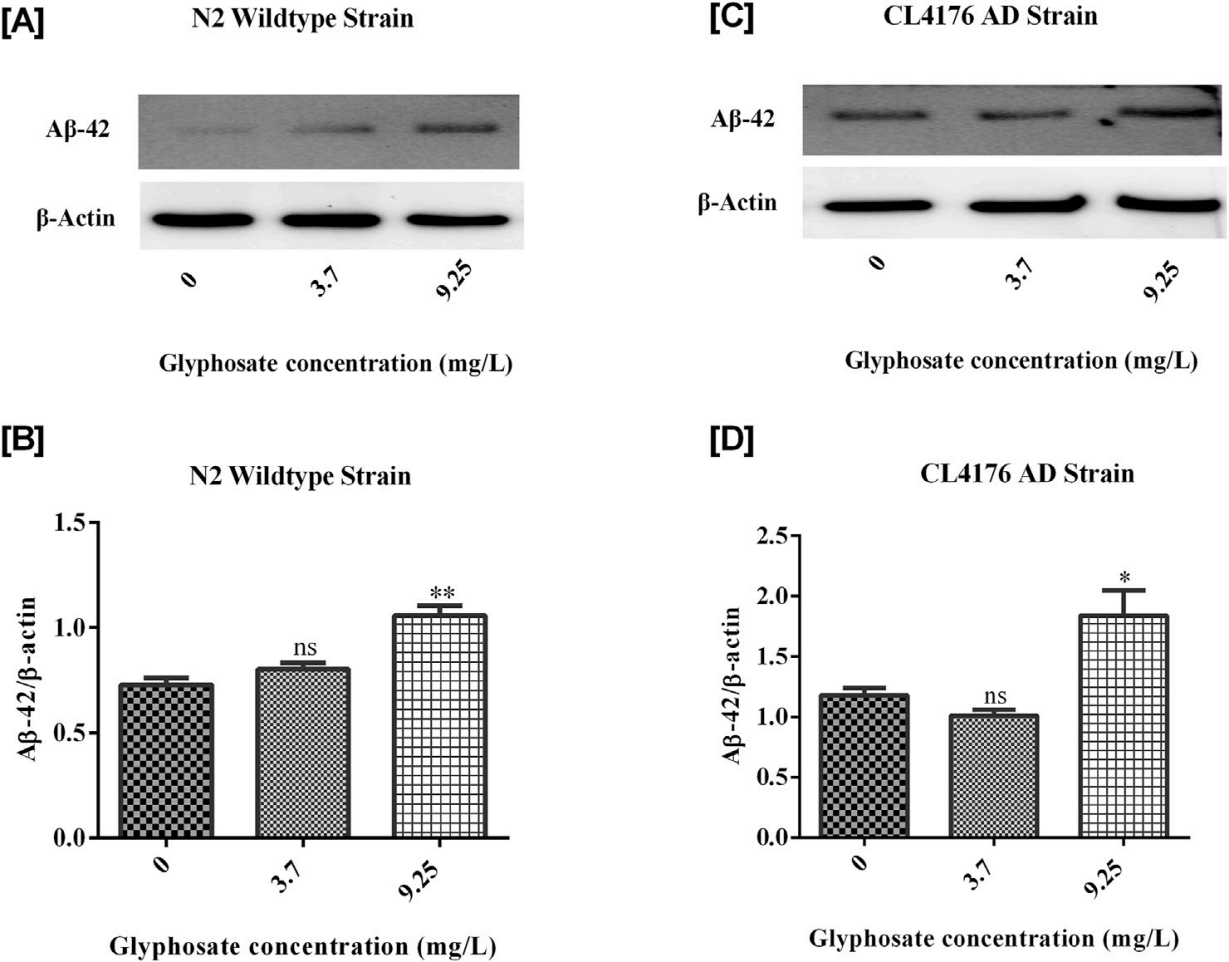
**(A)** Immunoblot of Abeta (1-42) in wildtype N2 strain of C. elegans. **(B)** Histogram shows Glyphosate upregulates Amyloid-beta (1-42). There was a significant (***P < 0.01) increase in the expression of Abeta (1-42) protein of higher concentration of Glyphosate in the C. elegans N2 strain compared with the control group. Error bars represent mean ± SEM, **P < 0.01. **(C)** Immunoblot of Abeta (1-42) in CL4176 AD strain of *C. elegans*. **(D)** Histogram shows Glyphosate upregulates Amyloid-beta (1-42). There was a significant (***P < 0.01) increase in the expression of Abeta (1-42) protein of higher concentration of Glyphosate in the *C. elegans* CL4176 strain compared with the control group. Error bars represent mean ± SEM, **P < 0.01

**FIGURE 10:**
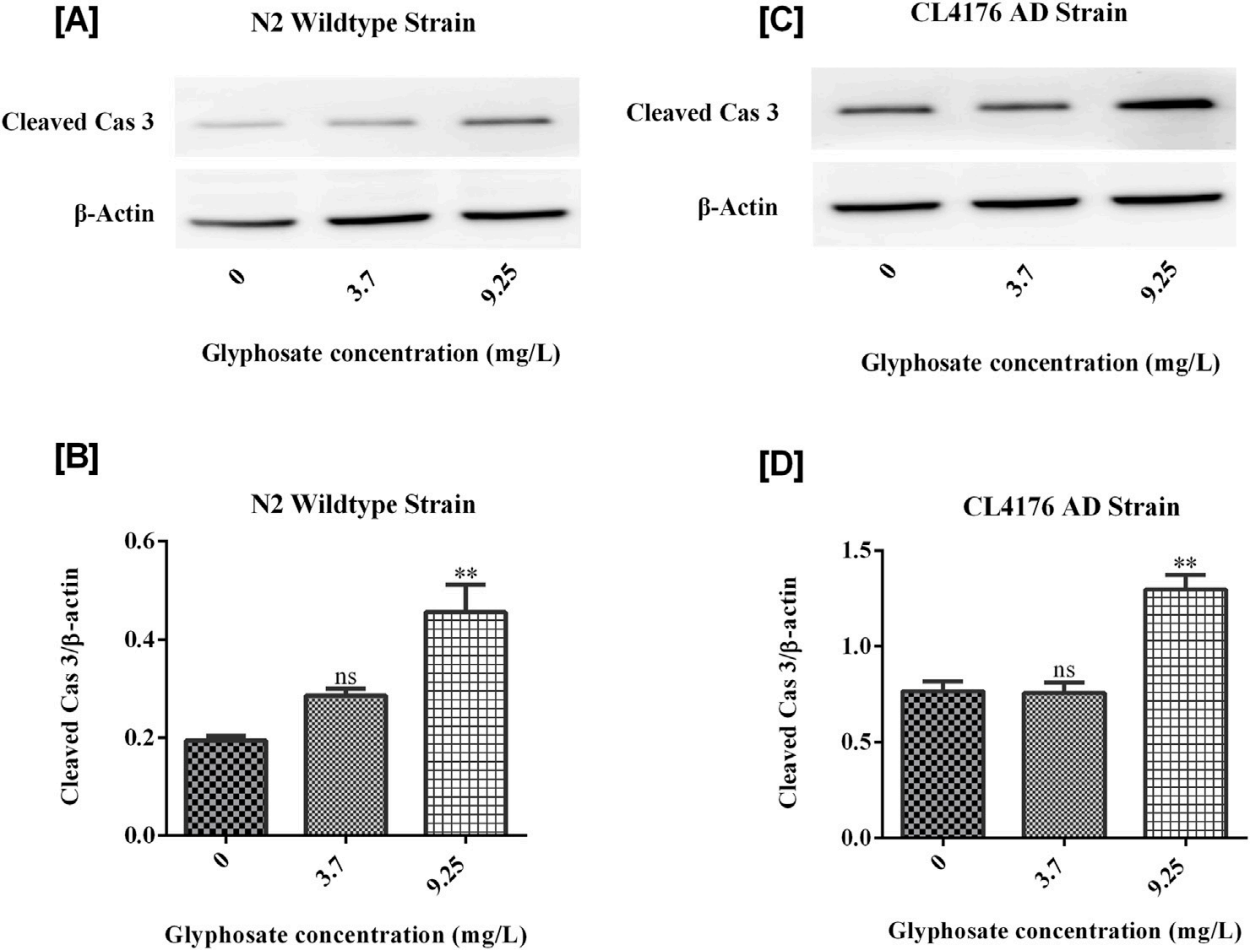
**(A)** Immunoblot of Cleaved Caspase 3 in wildtype N2 strain of *C. elegans*. **(B)** Histogram shows that Glyphosate upregulates Cleaved Caspase 3. There was a significant (P < 0.01) increase in the expression of Cleaved Cas 3 protein of higher concentration of Glyphosate in the *C. elegans* CL4176 strain compared with the control group. Error bars represent mean ± SEM, *P < 0.05. **(C)** Immunoblot of Cleaved Caspase 3 in CL4176 strain of *C. elegans*. **(D)** Histogram shows that Glyphosate upregulates Cleaved Caspase 3. There was a significant (P < 0.01) increase in the expression of Cleaved Cas 3 protein of higher concentration of Glyphosate inthe *C. elegans* CL4176 strain compared with the control group. Error bars represent mean ± SEM, *P < 0.05.

To investigate the neurotoxic effects of glyphosate on wild-type N2 and transgenic *C. elegans* AD models, we focused on neuronal cell death via apoptosis by analyzing cleaved caspase-3 protein. To understand why glyphosate may increase neuronal damage in CL4176, we measured Abeta (1-42) protein levels. All worms were collected after 24 h of induced paralysis, and parallel populations were prepared for Western blot analysis. As shown in Figures 9, 10, the group exposed to a high dose of glyphosate (9.25 mg/L) showed an increase in Abeta (1-42) protein and cleaved caspase 3 protein expressions compared to control N2 and AD models. These findings demonstrate that glyphosate accelerates neurotoxicity by upregulating cleaved caspase-3 and Abeta (1-42) proteins.

## 4 Discussion

Humans today are exposed to various toxic substances in the environment. Certain workers, particularly those in specific industries, face occupational exposure to these toxicants. Research has shown that these compounds can cause various harmful effects, from neurological disorders to genetic mutations (Thetkathuek et al., 2015). The implications of these findings are significant, as glyphosate is one of the most extensively used herbicides in the world. Given the widespread use of this chemical in agriculture and residential settings, the potential neurotoxic effects on human populations are concerning (Costas-Ferreira et al., 2022). Chronic exposure, even at low levels, could have cumulative effects on neurological health, particularly for vulnerable populations such as the elderly, who are already at risk for Alzheimer’s disease (Torres-Sánchez et al., 2023). The neurotoxic effects of glyphosate, a widely used herbicide, have gained increasing attention due to its potential role in accelerating neurodegeneration, particularly in models of AD (Bartholomew et al., 2024). In *C. elegans*, a well-established model organism for neurodegenerative diseases, exposure to glyphosate has been shown to exacerbate neuronal dysfunction, promote oxidative stress, and enhance amyloid-β (Aβ) aggregation—hallmarks of AD pathology (Jayasumana et al., 2015). These findings are consistent with mammalian studies, where glyphosate exposure has been linked to mitochondrial dysfunction and increased production of reactive oxygen species (ROS), contributing to neuronal death and cognitive deficits; (Mesnage et al., 2017; Ait-Bali et al., 2020). Notably, epidemiological studies suggest a correlation between long-term glyphosate exposure and increased risk of neurodegenerative diseases in humans, raising significant public health concerns (Fluegge et al., 2016; Evalen et al., 2024). The findings of this study suggest a strong link between glyphosate exposure and the exacerbation of Alzheimer’s disease-like symptoms in the model organism *C. elegans*. The concentration-dependent behavioral impairments observed in head thrash, body bending, body length, and pharyngeal pumping provide evidence of neurotoxicity, which is further corroborated by increased oxidative stress markers. Glyphosate’s effect on reducing catalase enzyme activity and promoting amyloid-beta (Aβ) accumulation in this model reinforces its potential role as an environmental factor contributing to neurodegenerative diseases, particularly Alzheimer’s (Bartholomew et al., 2024). Further research is necessary to investigate whether similar mechanisms of toxicity occur in mammals and, by extension, in humans. In particular, long-term studies focusing on chronic, low-dose exposure to glyphosate are needed to understand, the broader public health implications.

The *C. elegans* model provides a valuable *in vivo* system to dissect the molecular mechanisms underlying glyphosate-induced neurotoxicity, offering translational relevance to human AD pathology due to the conservation of key neurodegenerative pathways (Fang et al., 2019). These findings collectively underscore the urgent need for stricter regulatory measures on glyphosate use and further investigation into its long-term effects on human neurological health.

Additionally, future studies should explore potential interventions that could mitigate the neurodegenerative effects of glyphosate, possibly by targeting oxidative stress or amyloid-beta accumulation pathways. This study’s findings also raise questions about the regulatory standards governing glyphosate usage. Current guidelines primarily focus on its environmental and acute toxicological effects, but its potential role in long-term neurodegenerative diseases may necessitate stricter regulations or reconsideration of acceptable exposure levels. Moreover, these results underscore the importance of developing safer herbicides and encouraging agricultural practices that minimize reliance on potentially neurotoxic chemicals.

### 4.1 Limitations

While this study provides important insights into the potential neurotoxic effects of glyphosate using a *C. elegans* model, it has several limitations that should be acknowledged. The findings are based on a non-mammalian organism, which may not fully replicate the complexity of human neurological systems. Therefore, caution should be exercised when extrapolating these results to humans. Additionally, this study focused on short-term exposure under controlled laboratory conditions, which may not reflect real-world exposure scenarios. Future research using mammalian models and long-term, low-dose exposure studies is necessary to validate these findings and better understand the implications for human health.

## 5 Conclusion

**FIGURE 11:**
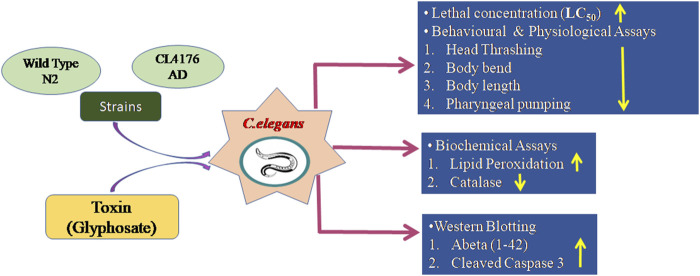
This figure represents that glyphosate has toxic effects in wild type N2 and CL4176 AD strains of *Caenorhabditis elegans* and causes behavioral, biochemical, and molecular changes in *Caenorhabditis elegans*.

In conclusion, the current results and recent mammalian studies, support the hypothesis that glyphosate exposure may influence Aβ peptide levels and contribute to AD-like pathology. These findings warrant further investigation into the implications for human health, especially considering the widespread use of glyphosate-based herbicides. Long-term studies focusing on chronic, low-dose exposure to glyphosate are necessary to understand, the broader public health implications. Additionally, future studies should explore potential interventions that could mitigate the neurodegenerative effects of glyphosate, possibly by targeting oxidative stress or amyloid-beta accumulation pathways as shown in Figure 11.

## Data Availability

The raw data supporting the conclusions of this article will be made available by the authors, without undue reservation.
